# Dog ownership during adolescence alters the microbiota and improves mental health

**DOI:** 10.1016/j.isci.2025.113948

**Published:** 2025-12-03

**Authors:** Eiji Miyauchi, Miku Yamaoka, Itsuka Kamimura, Mami Mizuta, Miya Takenaka, Uruma Akiyama, Masami Kawasumi, Nobuo Sasaki, Hiroshi Ohno, Shuntaro Ando, Syudo Yamasaki, Atsushi Nishida, Kazutaka Mogi, Miho Nagasawa, Takefumi Kikusui

**Affiliations:** 1 Institute for Molecular and Cellular Regulation, Gunma University, 3-39-15 Showa-machi, Maebashi, Gunma 371-8512, Japan; 2 Laboratory for Intestinal Ecosystem, RIKEN Center for Integrative Medical Sciences, 1-7-29 Suehiro-cho, Tsurumi-ku, Yokohama, Kanagawa 230-0045, Japan; 3 Department of Animal Science and Biotechnology, School of Veterinary Medicine, Azabu University, 1-17-71 Fuchinobe, Chuo-ku, Sagamihara-shi, Kanagawa 252-5201, Japan; 4 Department of Neuropsychiatry, Graduate School of Medicine and Faculty of Medicine, The University of Tokyo, Tokyo, Japan; 5 Unit for Mental Health Promotion, Research Center for Social Science ＆ Medicine, Tokyo Metropolitan Institute of Medical Science, 2-1-6 Kamikitazawa, Setagaya-ku, Tokyo 156-8506, Japan; 6 Center for Human and Animal Symbiosis Science, Azabu University, 1-17-71 Fuchinobe, Chuo-ku, Sagamihara-shi, Kanagawa 252-5201, Japan

**Keywords:** microbiome, psychology

## Abstract

Adolescents who own dogs have higher well-being than those who do not; however, it is unclear what the underlying mechanism explains how dog ownership affects adolescents’ well-being. As dog ownership influences the composition of the microbiota in the home environment, we examined the microbiome of dog-owning adolescents and analyzed associations with mental health and behavior in the teenage cohort participants (*n* = 345). Our findings reveal that dog-owning adolescents showed fewer problems with psychological scores, and some commensals were correlated with adolescents’ psychological scores. Mice treated with the microbiota of dog-owning adolescents showed a higher social approach to a trapped cagemate. An association analysis was conducted between the adolescents’ psychological scores and the mouse behavior with the abundance of each amplicon sequence variant (ASV) of the microbiome, and we found that ASVs belonging to *Streptococcus* were correlated with the social approach in ex-germ-free mice and mental scores in adolescents. These results suggest that microbiota may be partly involved in improving the well-being of adolescents living with dogs.

## Highlights

• Dog ownership in adolescence is linked to improved mental health
• Adolescents with dogs show distinct microbiota profiles
• Dog-related microbiota transfer enhances mouse social behavior
• *Streptococcus* ASVs correlate with well-being in adolescents and mice

## Graphical abstract

**Figure:**
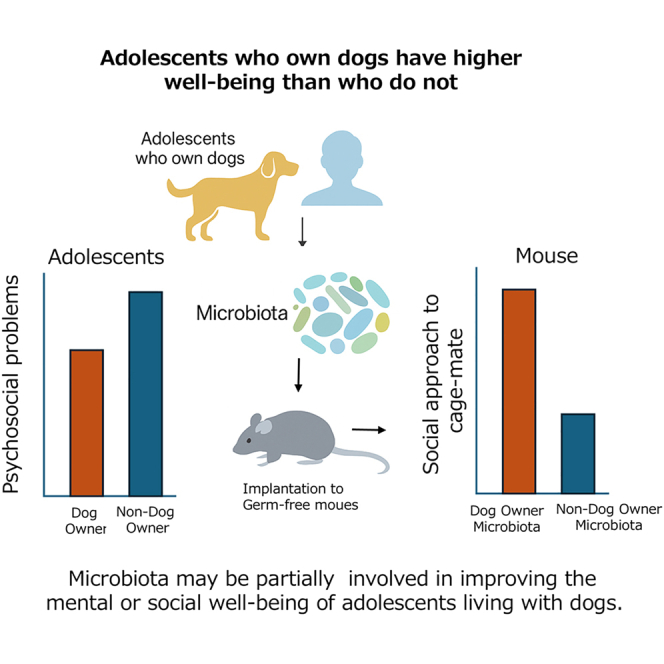

## Introduction

Adolescence is a critical period during which one’s life is shaped and influenced by one’s environment.^1^ During this period, social interactions and relationships, especially with family members, can have irreversible effects on mental and emotional development.^2^ Significant brain development occurs during adolescence and is associated with hormonal changes.^3^ This development takes place in areas of the brain like the limbic system, which is responsible for social behavior, processing rewards, and emotional reactions.^4^ Simultaneously, the prefrontal cortex, which is responsible for executive functions such as decision-making, social recognition, impulse control, and future planning, matures during adolescence.^5^ Thus, adolescence is crucial for the neurobehavioral development in humans.^4^

Dogs are the most popular companion animals globally, and their ownership has been reported to positively affect their owners’ mental and physical health.^6^ They can help alleviate loneliness, and spending time with them supports emotional well-being.^7^ Interacting with dogs reduces stress and anxiety, promotes relaxation, and fosters social interactions, strengthening social bonds.^8^ However, previous studies on this subject have reported conflicting results. For instance, the management of routine dog care, training, and disease can induce stress.^9^ A recent systematic review revealed a scarcity of high-quality longitudinal studies that accounted for potential effects of having a dog on human health.^10^

Several studies have suggested an association between pet ownership and mental well-being among adolescents.^11^^,^^12^ However, as many of these are cross-sectional and retrospective studies with limited longitudinal studies, further research is needed. In a longitudinal study of adolescents in Tokyo (Tokyo Teen Cohort), we found that dog ownership predicted and maintained mental well-being, whereas cat ownership predicted a decline in mental well-being from the 10–12 years of age.^6^ We also found that individuals who owned a dog at a young age and continued to own it later in life scored higher on measures of companionship and social support.^13^ These findings suggest that experiences of dog ownership during adolescence may be related to social outcomes later in life, such as increased companionship.

Various environmental factors, including dog ownership, can influence the composition of the human gut microbiota.^14^ A study comparing the microbial diversity of households with and without dogs found that the gut microbiota of dog owners differed from that of non-dog owners, with households with dogs exhibiting richer microbial diversity.^15^ For example, one study reported an increase in the abundance of *Bifidobacteriaceae* and *Ruminococcaceae* among dog owners.^14^ Furthermore, one study demonstrated that living with a dog in infancy can reduce the risk of allergic disorders.^16^ In addition, exposing allergy model mice to bacteria extracted from the house dust of dog-owning households provided protection against airway allergen challenges,^17^ suggesting that dog ownership can contribute to human health through bacterial exposure. For example, dog ownership modulates gut microbiota in elderly people.^14^ We recently revealed that small numbers of bacteria can be transferred between owners and their dogs, and vice versa.^18^ In humans, kissing facilitates the transfer of oral microbiota.^19^ Since kissing and licking are common in human-dog interactions, it is speculated that these interactions may alter the oral and gut microbiota of owners, potentially influencing gut-brain function.

Interestingly, social behavior in animals is modulated by the gut microbiome. Dysbiosis of the gut microbiota in developing mice decreases sociality and oxytocin responsiveness in the brain, which is one of the key neurotransmitters modulating social behavior.^20^ The effects of the microbiota on human sociability have also been reported (see review, Sherwi et al.^21^). One study demonstrated that human gut microbiota composition can predict social decision-making patterns.^22^ Recent evidence has highlighted the connection between the gut and brain, which is modulated by gut microbiomes through neural, neuroendocrine, and metabolic pathways.^21^ These pathways involve various neurotransmitters, hormones, cytokines, and bioactive metabolites, such as oxytocin,^23^ suggesting that the gut microbiome, especially during development, can shape the quantity and quality of social relationships in humans.^21^

This study hypothesizes that dog ownership affects adolescents’ microbiota, which in turn affects their mental and physical states. Specifically, we focus on the relationship between adolescents’ sociality and their microbiota. To clarify this causal relationship, we collected bacteria from adolescents who owned dogs, administered them to germ-free mice, and examined the effects of the microbiota on the social behavior of humanized ex-germ-free mice.

## Results

### Mental health and behavioral problems among dog-owning and non-dog-owning adolescents

**Figure 1:**
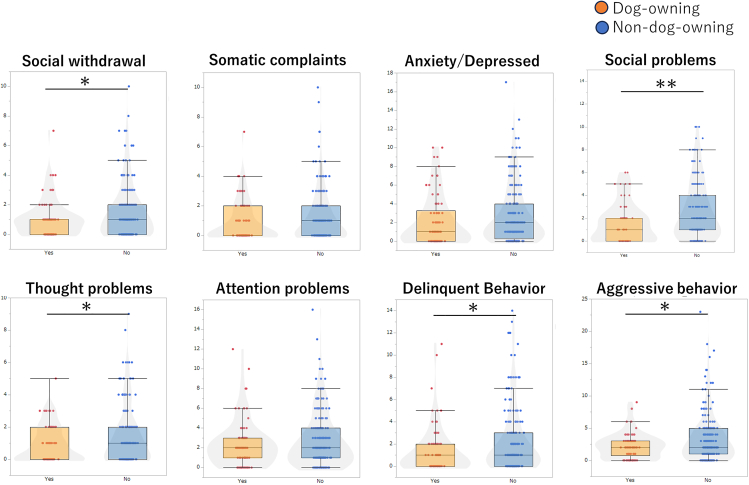
Mental health and behavioral problems among dog-owning and non-dog-owning adolescents CBCL subscale scores were compared between dog-owning and non-dog-owning adolescents. The analysis included participants for whom both pet ownership data at age 13 and CBCL scores at age 14 were available (*n* = 266; dog owners = 74, non-dog owners = 192). The subscales included social withdrawal, somatic complaints, anxiety/depressed, social problems, thought problems, attention problems, delinquent behavior, and aggressive behavior. Generalized linear models (GLMs) were used to examine group differences, both before and after adjusting for covariates (sex, annual household income, number of siblings, and number of family members). Scores for social withdrawal, social problems, thought problems, delinquent behavior, and aggressive behavior were significantly lower in dog-owning adolescents. ∗*p* < 0.05, ∗∗*p* < 0.01, by GLM followed by Mann-Whitney post hoc test.

To reveal the effect of dog ownership on mental status in adolescents, we analyzed the data from the participants with no missing data (*n* = 343) for the dog-owning status at age 13 and the Child Behavior Checklist (CBCL) subscales at age 14. The analyzed participants were 96 dog owners (male, 57; female, 39) and 247 non-dog owners (male, 139; female, 108) (Table S1). The dog-owning status of adolescents at age 13 predicted the CBCL subscales at age 14 as mental health and behavioral scores. The social problems score among dog-owning adolescents was significantly lower than that among non-dog-owning adolescents with the largest effect size (β = −0.192, *p* = 0.002). In addition, social withdrawal, thought problems, delinquent behavior, and aggressive behavior scores among dog-owning adolescents were lower than those among non-dog-owning adolescents (β = −0.122, −0.143, −0.133. −0.146, *p* = 0.048, 0.020, 0.030, 0.017, respectively; Figure 1). After adjusting demographic covariates (sex, annual household income, number of siblings, and number of family members), these differences still remain with the largest effect size of social problems (β = −0.194, *p* = 0.002).

### Microbiota correlated with behavior in human adolescents

**Figure 2:**
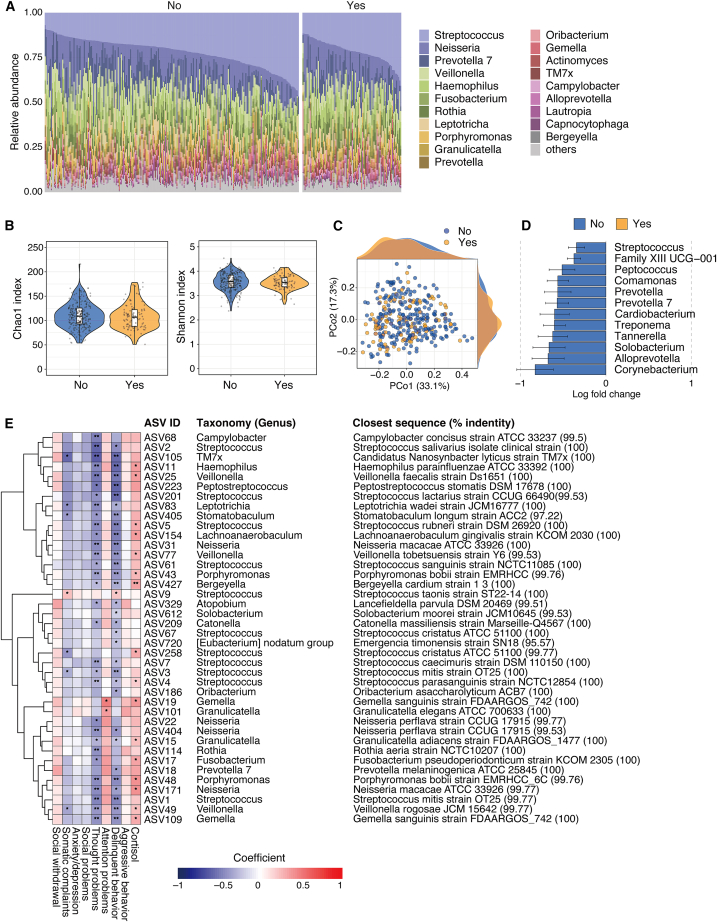
The effects of dog ownership on salivary microbiota (A) Bar plot representing the top 15 genera in the salivary samples of participants with (*n* = 96) or without dogs (*n* = 247); the remainder were labeled “others.” The *x* axis represents individual participant samples. (B) Chao1 index (left) and Shannon index (right) of the salivary microbiota. (C) Principal coordinate analysis of Bray-Curtis dissimilarity of the salivary microbiota. The density plots depict the sample distribution along the PCo1 and PCo2 axes. (D) Differentially abundant genera (adjusted *p* value < 0.05) between the groups analyzed by ANCOM-BC. (E) Heatmap showing the significant associations between behavioral and physiological scores and ASV abundance in the salivary samples analyzed by MaAsLin2. ASV identifier (ID), annotated genera, and the closest-matching strains are shown on the right ∗*p* < 0.05, ∗∗*p* < 0.01; MaAsLin2 (false discovery rate [FDR]-adjusted).

We conducted an analysis of salivary microbiota in non-dog-owning and dog-owning respondents. In total, 343 samples were sequenced, yielding 6,829,854 reads. At the genus level, the salivary samples were dominated by *Streptococcus* (21.9%), *Neisseria* (13.0%), and *Prevotella 7* (8.3%) (Figure 2A). The richness (Chao1 index) and evenness (Shannon index) of the salivary microbiota were comparable between groups (Figure 2B). Principal coordinate analysis based on Bray-Curtis dissimilarity also showed that the structures of the salivary microbiota did not differ between groups (Adonis, F = 1.6655, R^2^ = 0.00486, *p* = 0.097) (Figure 2C). Instead, the analysis of microbiome compositions with bias correction (ANCOM-BC) revealed some genera with differential abundances between the groups. Twelve genera, including the predominant genera *Streptococcus* and *Prevotella 7*, were significantly less abundant in the saliva of non-dog owners (Figure 2D). These results suggest that dog ownership affected the abundances of specific oral bacterial genera of their owners, while overall diversity and microbial community structure remained unchanged.

To determine whether oral bacteria were associated with behavioral and physiological scores, we applied MaAsLin2 (microbiome multivariable associations with linear models) to our dataset. This analysis revealed that the relative abundance of several amplicon sequence variants (ASVs) was significantly associated with the incidence of thought problems, delinquent behavior, attention problems, and cortisol concentration (Figure 2E). For example, ASVs belonging to the genus *Streptococcus* (ASV1, ASV2, ASV4, ASV5, and ASV7) were negatively associated with thought problems and delinquent behaviors. Thus, dogs may influence their owners’ thought problems and social behavior, at least in part, through changes in the microbiota.

### Mouse behavioral tests

**Figure 3:**
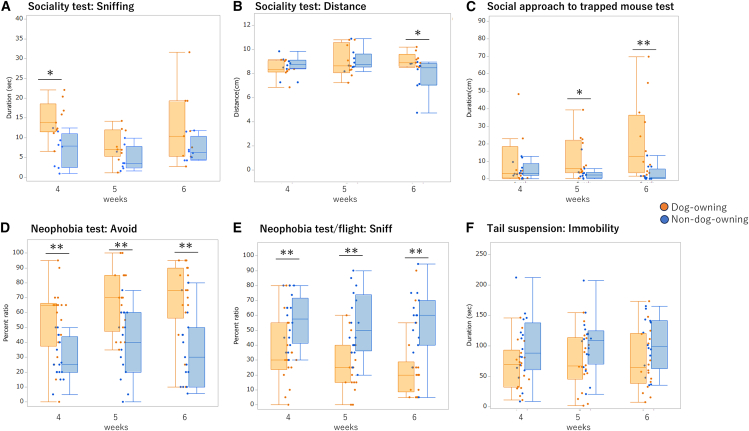
Behavioral differences between dog-owning and non-dog-owning groups of ex-germ-free mice (A and B) In the sociality test, the dog-owning group showed higher anogenital sniffing at 4 weeks of age and longer individual distance at 6 weeks of age compared to the non-dog-owning group (PERMANOVA followed by Mann-Whitney post hoc test). (C) The social approach to trapped mice test showed a longer social approach to trapped mice in the dog-owning group compared to the non-dog-owning group (PERMANOVA followed by Mann-Whitney post hoc test). (D and E) For the neophobia test, the dog-owning group showed higher avoidance behavior and lower sniffing behavior as compared to the non-dog-owning group (PERMANOVA followed by Mann-Whitney post hoc test). (F) In the tail suspension test, there was a group difference between dog-owning group and non-dog-owning group; however, there was no difference in the post hoc test. ∗*p* < 0.05, ∗∗*p* < 0.01, ∗∗∗*p* < 0.001; PERMANOVA followed by Mann-Whitney post hoc test.

To investigate whether changes in the oral microbiome are associated with alterations in social behavior among dog-owning adolescents, we transplanted their oral microbiota into germ-free mice and assessed the resulting social behaviors in the recipient animals. In the sociality test, anogenital sniffing was higher in the dog-owning group (Figure 3A; F(1,16) = 4.95, *p* < 0.05), and post hoc test revealed the group differences at 4 weeks old (*p* < 0.05). Individual distance in the sociality test was longer in the dog-owning group (Figure 3B; F(1,16) = 2.65, *p* < 0.05) and post hoc test revealed the group difference at 6 weeks (*p* < 0.05). In the social approach to the trapped mouse test, social approach behaviors were higher in the dog-owning group than in the non-dog-owning group (Figure 3C; F(1,23) = 4.32, *p* < 0.01) and post hoc test revealed the group difference at 5 and 6 weeks (*p* < 0.05 and *p* < 0.01, respectively). In the neophobia test, avoidance was higher (Figure 3D; F(1,33) = 15.6, *p* < 0.001) and sniffing behavior was lower in the dog-owning group at all ages (Figure 3E; F(1,33) = 12.7, *p* < 0.001). In the tail-suspension test, there was a group differences found (Figure 3F; F(1,33) = 3.32, *p* < 0.05), but there was no difference in the post hoc test. Other behavioral tests, such as the marble-burying, and aggression bites did not reveal group differences.

### Correlation analysis between human and mice behavior-microbiota

**Figure 4:**
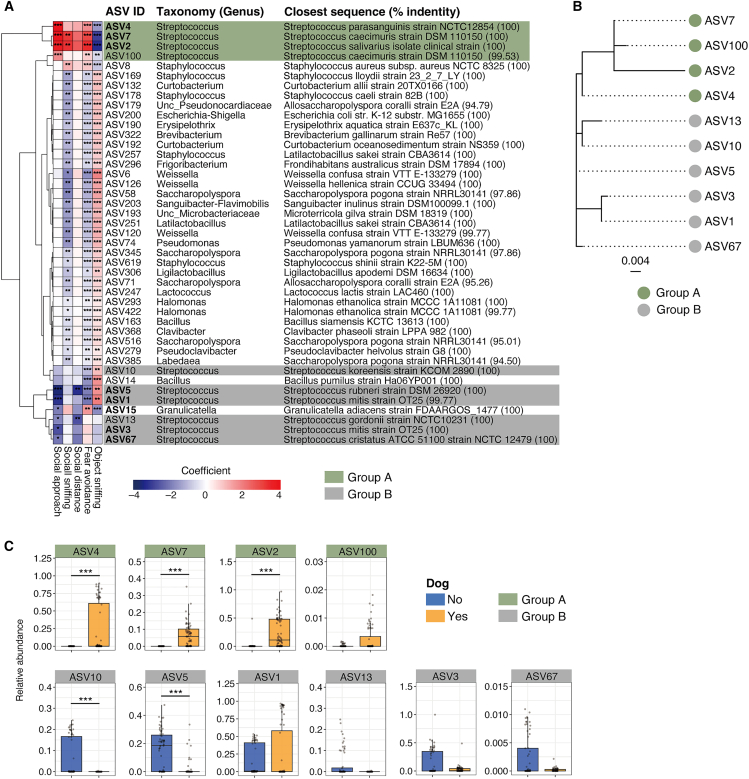
Associations between microbiota and behaviors in ex-germ-free mice; *n* = 17 mice (no) and *n* = 18 mice (yes), with three longitudinal fecal samples and behavioral scores collected from each mouse (A) Heatmap showing the associations between behavioral scores and ASV abundance in fecal samples of ex-germ-free mice transplanted with the participants’ salivary bacteria. ASVs showing significant associations with scores in both humans (Figure 1E) and mice are indicated in bold. *Streptococcus* ASVs were divided into two groups (groups A and B) based on their association patterns and are highlighted with different colors. (B) Phylogenetic tree based on the 16S rRNA gene sequences of *Streptococcus* ASVs. (C) Relative abundance of *Streptococcus* ASVs in the feces of ex-germ-free mice ∗*p* < 0.05, ∗∗*p* < 0.01, ∗∗∗*p* < 0.001; MaAsLin2 (FDR-adjusted) (A) and two-sided Wilcoxon test (C).

Next, we analyzed the fecal microbiota of the ex-germ-free mice. Corresponding to the salivary samples, *Streptococcus* was the most dominant genus in the feces of these recipient mice, accounting for 69.1% of the composition. MaAsLin2 analysis using fecal microbiota and behavioral test data demonstrated that several ASVs were associated with sociability indices (Figure 4A). Notably, *Streptococcus* ASVs were strongly associated with social approaches to trapped mice, although their association patterns varied among ASVs. According to the association pattern, *Streptococcus* ASVs were classified into two groups, designated as groups A and B, which displayed positive and negative associations with social approaches to trapped mice, respectively (Figure 4A). The phylogenetic tree of these ASVs also clearly separated them into groups A and B, implying that the *Streptococcus* strains in groups A and B were functionally distinct and may have had different effects on the host (Figure 4B). The relative abundance of these *Streptococcus* ASVs in the feces of ex-germ-free mice differed among the groups; that is, the ASVs in group A showed an increase in ex-germ-free mice inoculated with saliva obtained from dog owners, whereas those in group B exhibited a decrease (Figure 4C). In the saliva of the adolescent participants, no significant differences were observed in these ASVs between non-dog owners and dog owners (Figure S1), while the abundance of some ASVs such as ASV4 and ASV7 was associated with adolescent mental scores (Figure 2E). Taken together, these results imply that *Streptococcus* strains shared between with humans and dogs, such as ASV4, potentially affect the behavior of the host.

## Discussion

We reported in prior research that well-being is higher in dog-owning adolescents than in non-dog-owning ones. In this study, we found that dog-owning adolescents had lower scores on problems related to social relationships (social problems, social withdrawal, and delinquent behavior) and showed different microbiota compositions in saliva than non-dog-owning adolescents. We focused on microbiota to elucidate the underlying mechanisms of this finding. Adolescents’ bacteria were administered to germ-free mice and their social behavior was examined to elucidate the function of the microbiota. The results showed that mice treated with the bacteria from dog-owning adolescents exhibited increased social approaches toward trapped mice, which is related to pre-concern behavior.^24^ In addition, the sniffing behavior toward stranger mice increased. In microbiota analyses, ASV belonging to *Streptococcus* was similarly associated with the sociability of adolescents and ex-germ-free mice. ASV4 is negatively associated with delinquent behavior in adolescents, which represented antisocial behavior such as telling lies and insulting others. The same ASV was positively associated with pre-concern behavior in humanized mice. Although it is not possible to directly compare human and mouse behavior, these results suggest that the microbiota is partly responsible for the improvement in adolescents’ social behavior after living with dogs. While dogs’ positive effects on physical and mental health have been reported in previous literature,^8^ this is the first study to examine the involvement of the microbiome in the mental and behavioral well-being of dog-owning adolescents.

The characteristics of dog-owning adolescents identified in this study suggest enhancing social behavior skills, leading to stable and good family and friends’ relationships. Previous reports have described the associations between positive parent and adolescent relationships and high levels of self-esteem, life satisfaction,^25^ and overall happiness, as well as low levels of physical symptoms and emotional distress.^2^ Recent systematic review described that that friendship quality, which includes features such as companionship and social support is positively associated with subjective well-being,^26^ These studies suggest that social integration with family members and friends can enhance adolescents’ mental and behavioral health as well as their life satisfaction and happiness. Our findings of increase of social skills in dog- owning adolescents imply that dog ownership may influence adolescents’ social relationships with family and others and increase their well-being. One thing to note is that the Tokyo TEEN Cohort Project (TTC) is conducted in the Tokyo metropolitan area, where socioeconomic status is different from other areas in Japan. Further research is needed to determine whether the effects observed in dogs in this study are similar in other regions.

Growing evidence supports the role of gut microbiota-host neurobehavioral interactions in shaping social behaviors across a diverse range of animal species. For instance, supplementation of honeybees with a combination of *Bifidobacterium* and *Lactobacillus* has been shown to enhance cooperative behaviors, as indicated by increased hive productivity.^27^ Experimental studies using animal models have demonstrated that gut microbial communities can influence a range of neurobehavioral phenotypes, including stress reactivity,^28^ social anxiety, depression-like behaviors, and communicative functions.^29^ Notably, the administration of *Limosilactobacillus reuteri* has been shown to enhance activity in the brain oxytocin system and improve social behavior in mice.^30^ Additionally, interventional studies in humans have indicated that the modulation of the gut microbiome through symbiotic supplementation can promote prosocial behaviors, including increased fairness and cooperation in economic decision-making paradigms such as the Ultimatum Game.^22^ On the other hand, while research on the relationship between the human oral microbiome and social behavior is limited, there are correlations between the oral and gut microbiota.^31^ Recent studies have demonstrated the importance of microbiota in the oral cavity in social behavior and brain function in humans.^32^ For example, Wingfield demonstrated the association between depressive score and oral microbiome in adolescents.^33^ The present study demonstrated the importance of oral microbiota in humans by assessing psychological scores as well as the use of humanized gnotobiotic mice. The gut-brain connection is modulated by microbiomes through neural, neuroendocrine, immune, and metabolic pathways mediated by various bioactive signals.^21^ While we did not measure the molecules responsible for microbiota and social behavior in both adolescent and mouse experiments, one candidate was oxytocin, which is responsible for social connections, attachment, and buffering in animals.^34^ We found that oxytocin secretion increased in dogs and owners during interactions.^35^ Moreover, gut microbiota can modulate oxytocin neural activity via the vagal nerve.^20^^,^^30^ Together with these findings, it is important to measure oxytocin levels in both human and mouse models.

In this study, *Streptococcus* was associated with psychological scores in adolescents, and social behavior in mice. *Streptococcus* is a spherical bacterium, some species of which are endemic to the oral cavity, throat, and intestines of humans and animals. α-hemolytic *Streptococcus* was identified in approximately 17% of the oral cavities of healthy dogs.^36^ Another study demonstrated the possibility of *Streptococcus* transfer from dogs to humans.^37^ *Streptococcal* titer elevation was associated with exacerbation of obsessive-compulsive and tic disorders.^38^ Immunological changes by *Streptococcal* infection can change social behavior such as decrease of social investigation and increase of social avoidance in mice.^39^ However, in this study, certain *Streptococcus* ASVs were inversely associated with thought disorders, such as obsession, and delinquent behaviors, indicating that these ASVs may exert beneficial effects on the human host. In mouse models, the effects were contradictory; group A *Streptococcus* ASVs demonstrated a positive correlation with sociability, while group B *Streptococcus* ASVs exhibited a negative correlation with sociability. Similarly, in human samples, although several *Streptococcus* ASVs showed negative associations with behavioral problems, *Streptococcus* ASV9 showed a positive association, further suggesting functional diversity among strains within this genus. However, the analysis of the microbiota in this study was based on the 16S rRNA method, which does not capture full-length bacteria, and it is possible that the *Streptococcus* found in this study were different from those previously reported. Since some similar bacteria are known to behave differently, it is expected that the true function of *Streptococcus* found in this study will be clarified by the higher sequencing resolution and functional output provided by a shotgun metagenomic approach, allowing the field to address such associations in greater detail by identifying bacterial strains and their predicted metabolic byproducts in social behavior.

While it is not possible to make a direct comparison between human and mouse behavior, these results suggest that the microbiota is partly responsible for improvements in the social behavior of adolescents after they have lived with dogs. The difference in microbiome between dog-owning and non-dog-owning adolescents could be brought by living with dogs. Social interactions can enhance microbiome transmission in animals, which can fundamentally affect the costs and benefits of group living.^40^ In humans, social interactions can shape the microbiota via the horizontal transfer of bacteria.^41^ For instance, humans occupying the same environment share similar microbiota characteristics relative to those who do not, and this effect is greater when compared to genetic similarities.^42^ A recent shotgun metagenomic approach elucidated extensive bacterial strain sharing across individuals with distinct mother-to-infant, intrahousehold, and intrapopulation transmission patterns.^41^ This suggests that sharing a living environment is one of the most important factors for sharing oral and gut microbiome. In fact, we recently demonstrated the possibility of the direct transfer of gut bacteria between humans and dogs through human-dog cohabitation,^18^ a finding that was supported by other literature.^17^^,^^43^ This highlights the potential impact of the environment on horizontal microbial transmission and the differentiation of the gut microbiota between humans and dogs. While the microbiota in dogs was not examined in this study, dogs are mostly housed in rooms and share the same living environment with family members. Adolescents’ oral and gut microbiomes can be modulated by living with dogs, and this modulation can change adolescents’ social characteristics. Dogs are the oldest domesticated animals in human society. Consequently, social interactions during this long period of cohabitation may enable the preservation of gut microbial diversity across large periods of time, which may have important implications for the evolution and ecology of both human and dog microbiota.^44^ Another possibility is that living with dogs can change the physical and mental status of the adolescent, and these changes result in changes in the microbiota. Being accompanied by dogs can reduce stress responses and decrease cortisol secretion.^45^ Changes in cortisol and physical stress status can modulate the gut microbiome.^46^ In this context, the changes observed in humanized mice are thought to reflect changes in the gut microbiota caused by physical and psychological changes in humans, rather than the result of shared gut microbiota between humans and dogs. By tracking changes in endocrine stress levels, psychological measures, and gut microbiota over time in individuals who have started keeping dogs, it may be possible to identify temporal causality.

This study identified one of the potential mechanisms underlying the positive effects of dog ownership in adolescents. These effects were investigated psychologically and microbiologically, and changes in the microbiota observed in dogs owners during adolescence were shown to alter social behaviors in humanized mice. It is expected that selective culture and the administration of candidate bacteria will demonstrate whether the associated bacteria contribute to adolescents’ mental health. Longitudinal research using this TTC cohort should be conducted to determine the duration of persistence of the effects of increased prosociality due to dog ownership and the microbiota.

### Limitations of the study

There are several limitations in this study. First, the microbiota samples were collected from different sites in humans and mice—oral samples from adolescents and fecal samples from mice—making direct comparisons between human and mouse microbiota difficult. This was due to practical constraints during the participant visits, where only saliva could be collected. Given the technical difficulty of analyzing oral microbiota in mice and the growing evidence that oral microbes can translocate to the gut and modulate host physiology via the oral-gut axis,^47^ we focused on gut microbiota in recipient mice, although salivary samples were analyzed in humans. Future studies should consider collecting fecal samples from human participants to better assess the gut microbiota composition. Second, we used 16S rRNA sequencing, which has a limited taxonomic resolution and cannot distinguish between functionally distinct bacterial strains within the same genus. To better identify bacterial strains and their functional contributions, shotgun metagenomic sequencing is recommended. Third, we could not determine the causal factors of the changes in the microbiota in dog-owning adolescents. One potential explanation is that domestic dogs introduce unique microbial communities into the home environment, which in turn may influence the oral microbiota of adolescent cohabitants. Further research is needed to elucidate the direct and indirect effects of pet-associated microbial exposure on human microbiome dynamics. Finally, there are concerns about the generalization and consistency of mental scores obtained from adolescents. Although our cohort included individuals with a wide range of socioeconomic backgrounds and was designed to over-sample lower-income households, it is possible that the overall socioeconomic status of the participants—being based in the Tokyo metropolitan area—is slightly higher than that of the general population in Japan. Hence, despite adjusting for household income in our analysis, we cannot fully exclude the possibility that the relatively higher socioeconomic status may have influenced behavioral characteristics, such as reduced delinquency or enhanced social behavior. This may limit the generalizability of our findings to other populations with different socioeconomic profiles. Also, psychological assessments of adolescent participants were conducted only at the age of 14 years, leaving the longitudinal stability of these measures uncertain. Additionally, in the mouse model, social behaviors such as social sniffing and approach toward caged conspecifics were not consistently observed across different age groups. The interpretation of social sniffing and approach behaviors toward caged conspecifics in mice remains controversial. These behaviors may reflect prosocial tendencies, such as affiliative motivation or empathy-like responses, or simply indicate nonspecific social interests or investigatory behavior. These findings raise important questions about the sustainability and temporal consistency of the observed effects, warranting further investigation through long-term, cross-sectional, and developmental studies. Future studies should incorporate hormonal and neural activity measurements to verify the molecular mechanisms underlying microbiota-mediated changes in sociability.

## Resource availability

### Lead contact

Further information and requests for resources should be directed to and will be fulfilled by the lead contact, Takefumi Kikusui (kikusui@azabu-u.ac.jp).

### Materials availability

This study did not generate new unique reagents.

### Data and code availability

• 16S rRNA gene sequencing data reported in this study were deposited in the DNA Data Bank of Japan's BioProject (DRA018752) and other datasets are available from the corresponding author on request.
• This study did not generate code.
• Any additional information required to reanalyze the data reported in this paper is available from the lead contact upon request.

## Acknowledgments

We would like to sincerely thank all of the adolescents and their primary caregivers who participated in TTC. We acknowledge the work of all the interviewers who conducted the data collection. This study was financially supported by 10.13039/501100020959JST-Mirai Program, Japan, grant no. JPMJMI21J3, JST RISTEX grant no. JPMJRS24K1 and JSPS-KAKENHI (23H05472 to T.K. and 21H03333 and 21H05173 to M.N.).

## Author contributions

Conceptualization, E.M., H.O., S.A., S.Y., A.N., K.M., and T.K.; methodology, E.M., I.K., U.A., H.O., S.A., S.Y., A.N., and T.K.; formal analysis, E.M. and S.Y.; investigation, E.M., M.Y., I.K., M.M., M.T., U.A., S.Y., and T.K.; writing – original draft, E.M. and T.K.; writing – review and editing, N.S., H.O., S.A., S.Y., A.N., K.M., and T.K.; resources, E.M., N.S., H.O., S.A., S.Y., and A.N.

## Declaration of interests

All authors declare no conflict of interest.

## STAR★Methods

### Key resources table

**Table:** 

REAGENT or RESOURCE	SOURCE	IDENTIFIER
**Antibodies**		

Anti-Cortisol antibody	abcam	Cat# ab1949; RRID: AB_302703
Mouse IgG-Fc Fragment Antibody	Bethyl Laboratories, Inc.	Cat# A90-131A; RRID:AB_67172

**Biological samples**		

Human Saliva	This paper	
Human Urine	This paper	
Mouse Feces	This paper	

**Chemicals, peptides, and recombinant proteins**		

Cortisol, HRP Conjugate	MyBioSource.com.	Cat# MBS342079
Cortisol	Fujifilm WAKO chemicals	Cat# 088-02483
TMBZ	Fujifilm WAKO chemicals	Cat# 346-04031
BSA	Sigma-Aldrich	Cat# A7030
Lysozyme	Fujifilm WAKO chemicals	Cat# 120-02674
Achromopeptidase	Fujifilm WAKO chemicals	Cat# 019-28531
Proteinase K	Fujifilm WAKO chemicals	Cat# 166-28913
Phenol-chloroform	Nacalai tesque	Cat# 25970-56
KAPA HiFi Hot Start Ready Mix	KAPA Biosystems	Cat# KK2602
AMPure XP Reagent	Beckman Coulter	Cat# A63881

**Critical commercial assays**		

Nextera XT Index Kit v2 Set A	Illumina	FC-131-2001
Nextera XT Index Kit v2 Set D	Illumina	FC-131-2004
MiSeq Reagent Kit v3	Illumina	MS-102-3003

**Deposited data**		

16S rRNA gene sequencing data reported in this study	DNA Data Bank of Japan's BioProject	DRA018752

**Experimental models: Organisms/strains**		

Mouse C57BL/6NCr	Nippon Clare Co., Ltd	https://www.clea-japan.com/products/other_disease/item_a0300

**Oligonucleotides**		

See Table S2 for oligonucleotide list	This paper	Table S2

**Software and algorithms**		

BORIS v.7.12.2	(Friard, O. & Gamba, M)	https://www.boris.unito.it/
JMP v.13.	JMP Statistical Discovery LLC.	https://www.jmp.com/ja/home
R v.4.2.3	CRAN	https://cran.r-project.org/
RStudio	RStudio Team	http://www.rstudio.com
R package “dada2” v.1.26.0	Callahan et al.	https://github.com/benjjneb/dada2
R package “phyloseq” v.1.42.0	Paul et al.	https://joey711.github.io/phyloseq/
R package “ANCOMBC” v.2.0.2	Lin et al.	https://www.bioconductor.org/packages/release/bioc/html/ANCOMBC.html
R package “MaAsLin2” v.1.12.0	Mallick et al.	https://huttenhower.sph.harvard.edu/maaslin/
R package “DECIPHER” v.2.26.0	Erik Wright	https://www2.decipher.codes
R package “phangorn” v.2.11.1	Schliep et al.	https://klausvigo.github.io/phangorn/
R package “ggplot2” v.3.4.4	Wickham et al.	https://ggplot2.tidyverse.org/index.html
R package “cowplot” v.1.1.1	Claus O. Wilke	https://cran.r-project.org/web/packages/cowplot/index.html
R package “pheatmap” v.1.0.12	Raivo Kolde	https://cran.r-project.org/web/packages/pheatmap/index.html
R package “ggtree” v.3.6.2	Yu et al.	https://github.com/YuLab-SMU/ggtree
R package “vegan” v.2.6-4	Oksanen et al.	https://github.com/vegandevs/vegan

### Experimental model and study participant details

#### Human participants

This study was conducted as a part of a population-based biomarker subsample study of the Tokyo TEEN Cohort Project (pb-TTC: http://ttcp.umin.jp), an ongoing, prospective, population-based birth cohort study of adolescents and their primary caregivers (mainly mothers) living in the Tokyo metropolitan area that investigates adolescents' health and development.^48^ Pb-TTC was a semi-structured study conducted from 17^th^ March 2017 to 17^th^ September 2018, and 345 participants of which were recruited from a larger sample of the TTC project. Participants in the pb-TTC were enrolled from a larger sample of the TTC projects. We did not set any exclusion criteria. At the first point of the TTC, a sample of 3171 households with adolescents aged 10 years (born between September 2002 and August 2004) was randomly selected from the basic resident register of three municipalities in Tokyo, Japan (Chofu, Mitaka, and Setagaya). At the second time point of the study, when the adolescents were 12 years old, 3007 households participated (follow-up rate: 94.8%). The adolescents’ primary caregivers provided written informed consent in the presence of trained interviewers. We collected longitudinal data at one-year intervals in sub-sample study, including anthropometric data (height, weight, and grip), information about the children’s pet ownership, and their mental health status. Urine was collected in the morning for cortisol assay and saliva samples for microbiome analysis were obtained from the participants when they visited the facility for detailed research. For the cortisol assay, the first urine sample was collected in the morning by the participants, and the samples were brought to the facility. The samples were stored at -20°C until the assay. Cortisol levels were measured by Air Plans-Bio, Co., Ltd. (Tokyo, Japan), which was contracted for the analysis.

The authors assert that all procedures that contributed to this work comply with the ethical standards of the relevant national and institutional committees on human experimentation and with the Helsinki Declaration of 1975, as revised in 2008. The study protocol for the TTC project was approved by the institutional review boards of the Tokyo Metropolitan Institute of Medical Science (approval number: 12–35), Graduate University for Advanced Studies (2012002), University of Tokyo (10057), and Azabu University (#57). Written informed consent was obtained from all the parents of the participating children, and informed assent was obtained from the children. The reporting of this study follows the Strengthening the Reporting of Observational Studies in Epidemiology (STROBE) guidelines.

#### Mouse models

Twelve male C57BL/6NCr germ-free mice (Nippon Clare Co., Ltd., Tokyo, Japan) were brought into an isolator (Sanki Kagaku Kogei Co., Ltd., Jic Co., Ltd., Tokyo, Japan) at three weeks of age and were kept in plastic cages (182 × 260 × 128 mm) with three mice per cage. The animals were fed γ-irradiated feed CMF (Oriental Yeast, Tokyo, Japan). The mouse experiments protocol was approved by the Animal Experiment Committee of Azabu University (No. 210325-15), which follows ARRIVE guideline.

### Method details

#### Dog-owning status, mental health, and well-being of adolescents

Semi-structured interviews were conducted with the adolescent participants to determine whether they had any pets at home using the question, “Do you have any pets?” at age 12-13. Their responses were coded into two dichotomized variables: A) owned (1) or not owned (0) for dogs. In this study, groups were divided based solely on whether or not adolescents owned dogs, and other pets were not considered. In addition, a detailed questionnaire on dog ownership was administered to determine how much time they spent interacting with dogs and their involvement in pet care.

Mental health and behavioral scores were measured using the Child Behavior Checklist (CBCL). The CBCL has eight subscales, including scores reflecting a child’s sociality (social withdrawal, somatic complaints, social problems, thought problems, attention problems, delinquent behavior, and aggressive behavior). The outcome variables in our study were derived from the Japanese version of the Child Behavior Checklist (CBCL; Achenbach et al. 1991^49^; Itani et al., 2001^50^), a caregiver-reported questionnaire designed to assess problematic behaviors in children. The original CBCL consists of 118 items; however, to reduce participant burden, we utilized a shortened version comprising 84 items. Each item was rated on a three-point scale: “not true,” “somewhat or sometimes true,” and “very true or often true.” From these responses, eight subscale scores were calculated: Social Withdrawal (9 items), Somatic Complaints (9 items), Anxiety/Depressed (14 items), Social Problems (8 items), Thought Problems (7 items), Attention Problems (11 items), Delinquent Behavior (13 items), and Aggressive Behavior (20 items). The subscale scores were obtained by summing the corresponding item scores, with higher scores indicating greater levels of behavioral or emotional problems”. The details of these scores were published in our previous reports.^51^

#### Mouse experiment procedures

The microbiome samples used were extracted from the TTC saliva samples of adolescents aged 12-13 years at the time of the research. After sampling, we refrigerated the saliva, from which a glycerol stock was prepared and stored frozen at -80°C. Saliva from 6 male adolescents (3 in each group) with representative psychological scale scores and microbiome composition in the dog-owning and non-dog-owning groups was selected for administration into the stomach using a gastric sonde, and the mouse tests were repeated three times.

For bacterial solution administration, saliva from both groups of adolescents was thawed on ice and centrifuged at 12,000 rpm for 15 minutes at 4°C. The supernatant was discarded and the sample was suspended in 200 μL of sterile saline to form a pellet. Each suspension was administered orally to 100 μl of 4-week-old GF mice. Mouse experiments were conducted three times during different periods, and the samples used in each experiment were from different adolescents.

Mouse fecal samples were collected at 4, 5, and 6 weeks of age and stored at -80°C in glycerol stock until the microbiome assay.

#### Mouse behavioral tests

The behavioral test battery was conducted among the mice three times at 4, 5, and 6 weeks of age in each mouse. Each battery contained a 2-day test. On day 1, the marble-burying test and neophobia test were conducted. On day 2, the sociality test, aggression bite, social approach to trapped mouse test, and tail-suspension test were conducted.

##### Sociality test

Two unfamiliar testing mice were selected and placed in an 82 mm × 260 mm × 128 mm plastic cage for 30 min of video recording. The average distance between two individuals was analyzed using a video-tracking system (Ethovison 13, Nordus, Netherlands), and the total duration of the following behaviors was measured for 30 min: allo-grooming, anogenital sniffing, chasing, head sniffing, and body-body contact.^52^

##### Social approach to trapped mouse test

When a mouse was presented with a trapped conspecific, they showed approach and sniffing behavior toward it,^53^ which was thought to be related to empathetic behavior, similar to pre-concern behavior in humans.^54^ The trapped mouse was confined in 50-mL polypropylene conical tubes with three air holes drilled in the sides and four holes drilled at the end. The tube was placed in the home cage of the testing mouse (82 × 260 × 128 mm), and behavior was video-recorded for 5 min. The total duration of chewing/sniffing/nose-in-the-hole behavior towards the tube with trapped mice was recorded.

##### Neophobia test

Mice often show neophobic responses such as aggressive or avoidant behavior towards novel inanimate objects.^55^ The methods were modified due to the limitation of the space of the isolator. The mouse was placed in a plastic cage (82 mm × 260 mm × 128 mm) and presented with aluminum foil wrapped around 300 mm tweezers for 5 s once every 15 s for 5 min, and avoidance (retreating from the object, increased distance from the object, turning the face away from object), sniffing, ignoring (no reaction), and aggression bites were measured. The behavioral score was calculated as the percentage of each behavior in the total behavioral duration.

##### Marble-burying test

Rodent marble-burying behavior in the marble-burying test is employed as a model or measure to study anxiety- and compulsive-like behaviors.^56^ Mice were placed in plastic cages (82 mm x 260 mm x 128 mm) and acclimated for 5 min on a 3 cm thick bedding. The mouse was removed from the cage once for 5 minutes and then returned to the cage with 12 stainless steel silver balls (ø9.52) placed on the bedding. Their behavior was recorded for 25 min and subsequently analyzed. The behavioral scores were determined based on the number of silver balls the surfaces of which were more than 2/3 hidden at the end of the test session and the distance they had traveled in the cage.

##### Aggression bites

When mice were presented with novel steel rods while restrained, they vigorously bit the rods that emerged beneath their chin. This aggressive biting behavior was positively correlated with the duration of social isolation, which is known to enhance aggression.^57^ The mouse was confined in a 50-mL polypropylene conical tube with three air holes on the sides and four on the tip and presented with tweezers in front of its face once every 15 s for 5 min. The ratio of behaviors including ignoring, holding, or biting the tweezers to the total behavioral responses was calculated.

##### Tail-suspension test

This test was used for assessing depressive-like behavior in mice.^58^ The tail of each mouse was clipped 30 cm above the floor. The mouse’s immobility time was monitored for 6 min.

#### Microbiota analysis

Human saliva was collected during a visit to the facility to participate in a detailed research study and immediately stored in sterile tubes at 4°C, then frozen at -80°C until the DNA extraction. Bacterial DNA was isolated using a Fast DNA Spin Kit (MP Biomedicals). The V3-V4 region of the 16S rRNA gene was amplified by PCR using the 341F/805R universal primers (Table S2). The resulting amplicons were indexed by eight additional cycles of PCR using the 2nd PCR primers (2ndF/2ndR, shown in Table S2). The pooled amplicons were quantified and qualified using the Qubit dsDNA HS Assay Kit (Thermo Fisher Scientific, Waltham, MA, USA) and High Sensitivity NGS Fragment Analysis Kit (Advanced Analytical Technologies), and then sequenced on a MiSeq (Illumina, 2 × 300-bp paired-end reads).

The mice’s fecal samples were collected before the behavioral tests and immediately stored in sterile tubes at -80°C until the DNA extraction. The samples were treated with lysozyme, achromopeptidase, and proteinase K,^59^ and the bacterial DNA was purified using a phenol-chloroform/isoamyl alcohol (25:24:1) solution. The amplicons of V3-V4 region, amplified as described above, were indexed using a Nextera XT index kit v2 (Illumina). The library was quantified and qualified using a KAPA library quantification kit (Roche) and an Agilent TapeStation (Agilent), and then sequenced on a MiSeq. The sequencing depth for human saliva samples totaled 6,829,854 reads, with a median of 19,312 reads per sample (minimum: 10,333; maximum: 39,795). For mouse fecal samples, the total was 7,204,762 reads, with a median of 53,627 reads per sample (minimum: 18,901; maximum: 92,115).

### Quantification and statistical analysis

While 343 participants had complete data at baseline (age 13 year), only 266 participants completed the CBCL at the one-year follow-up. Therefore, a generalized linear model analyses were conducted using data from 266 participants who provided both baseline and follow-up data, for examining whether dog ownership status predicted adolescents’ mental health (social withdrawal, somatic complaints, anxiety/depressed, social problems, thought problems, attention problems, delinquent behavior, aggressive behavior) after a year by adjusting for covariates. To control for potential confounding factors in human analyses, we included sex, annual household income, number of siblings, and number of family members as covariates in the regression models. These variables were selected based on their established associations with adolescent behavioral and psychological outcomes. Generalized linear models were used to assess the association between dog ownership and CBCL subscale scores, both with and without adjustment for these covariates.

For behavioral comparisons between the ex-germ-free mice of the dog-owning and non-dog-owning groups, the non-parametric MANOVA test (PERMANOVA) was conducted at each parameter using R statistics; group and week of testing were factors, due to lack of normal distribution. Post-hoc test was conducted at each age using Mann-Whitney non-parametric test. Statistical analyses of microbiome-phenotype associations were conducted with the MaAsLin2 package^60^ using R statistics.

The 16S rRNA reads were processed with DADA2 package (v1.26.0) in R to generate amplicon sequence variants (ASVs) according the DADA2 pipeline v1.8.^61^ The ASVs were taxonomically classified using the SILVA database v138.1. Downstream analyses, including alpha and beta diversity, were performed using the phyloSeq package (v1.42.0). Genera showing significantly different abundances between the groups were analyzed using the ANCOMBC package (v2.0.2) with default parameters. ASVs present in more than 20% of samples were used to investigate their association with host data using the MaAsLin2 package (v1.12.0). The sequences of *Streptococcus* ASVs were aligned using the DECIPHER package (v2.26.0), and a neighbor-joining tree was constructed using the phangorn package (v2.11.1). The data were visualized using ggplot2 (v3.4.4), cowplot (v1.1.1), pheatmap (v1.0.12), and ggtree (v3.6.2) packages.

### Additional resources

This manuscript was pre-registered in the following URL https://www.researchsquare.com/article/rs-5027712/v1.
