# Supplementary material for: Dog ownership during adolescence alters the microbiota and improves mental health
Source: iScience. 2025 Dec 3;28(12):113948. doi: 10.1016/j.isci.2025.113948 (PMC12767183; doi:10.1016/j.isci.2025.113948)
Supplement: Document S1. Figure S1 and Tables S1 and S2 [file mmc1.pdf]

iScience, Volume *28*

Supplemental information

Dog ownership during adolescence alters the microbiota and improves mental health

Eiji Miyauchi, Miku Yamaoka, Itsuka Kamimura, Mami Mizuta, Miya Takenaka, Uruma Akiyama, Masami Kawasumi, Nobuo Sasaki, Hiroshi Ohno, Shuntaro Ando, Syudo Yamasaki, Atsushi Nishida, Kazutaka Mogi, Miho Nagasawa, and Takefumi Kikusui

Supplemental Materials

Dog ownership during adolescence alters the microbiota and improves mental health

Eiji Miyauchi,[1,2] Miku Yamaoka,[3] Itsuka Kamimura,[3] Mami Mizuta,[3] Miya Takenaka,[3] Uruma Akiyama,[3] Masami Kawasumi,[2] Nobuo Sasaki,[1] Hiroshi Ohno,[2] Shuntaro Ando,[4] Syudo Yamasaki,[5] Atsushi Nishida,[5] Kazutaka Mogi,[3,6] Miho Nagasawa,[3,6] and Takefumi Kikusui.*[3,6]

*Correspondence: Takefumi Kikusui, PhD, DVM
Department of Animal Science and Biotechnology, School of Veterinary Medicine, Azabu University, 1-17-71 Fuchinobe, Chuo-ku, Sagamihara-shi, Kanagawa 252-5201, Japan
Tel/fax +81-42-756-1853
Email: Kikusui@azabu-u.ac.jp

Table S1 Baseline characteristics of participants (N=343)

	non dog owning (n=247, 72.0%)	dog owning (n=96, 28.0%)	p
Age in years, mean [s.d.]	13.93 [0.50]	13.87 [0.45]	0.324
Females, n (%)	108 (43.7)	39 (40.6)	0.603
Family annual income, n (%)			
Less than 4 million yen	20 (8.1)	9 (9.4)	0.299
4-6 million yen	65 (26.3)	17 (17.7)	
7-9 million yen	56 (22.7)	20 (20.8)	
More than 10 million yen	92 (37.2)	44 (45.8)	
Missing	14 (5.7)	6 (6.3)	
Number of siblings, mean [s.d.]	2.1 [0.9]	1.9 [0.7]	0.166
Number of family members, mean [s.d.]	4.1 [1.0]	4.0 [0.9]	0.242

Table S2: Primer information used in 16S RNA analysis

Primers	Sequences
341F	ACACTCTTTCCCTACACGACGCTCTTCCGATCTCCTACGGGNGGCWGCAG
805R	GTGACTGGAGTTCAGACGTGTGCTCTTCCGATCTGACTACHVGGGTATCTAATCC
2ndF	AATGATACGGCGACCACCGAGATCTACAC-Index2-ACACTCTTTCCCTACACGACGC
2ndR	CAAGCAGAAGACGGCATACGAGAT-Index1-GTGACTGGAGTTCAGACGTGTG

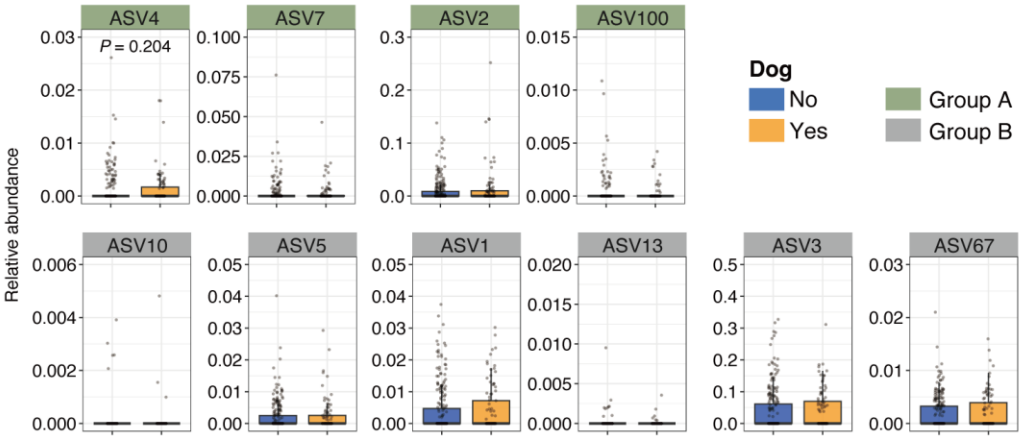

Supplementary Fig. S1. Relative abundance of Streptococcus ASVs in the saliva of the adolescents.
